# The Efficiency of Brain‐Derived Neurotrophic Factor Secretion by mRNA‐Electroporated Regulatory T Cells Is Highly Impacted by Their Activation Status

**DOI:** 10.1002/eji.202451005

**Published:** 2024-12-19

**Authors:** Jasper Van den Bos, Ibo Janssens, Morgane Vermeulen, Amber Dams, Hans De Reu, Stefanie Peeters, Carole Faghel, Yousra El Ouaamari, Inez Wens, Nathalie Cools

**Affiliations:** ^1^ Laboratory of Experimental Hematology Vaccine and Infections Disease Institute (VAXINFECTIO) Faculty of Medicine and Health Sciences University of Antwerp Antwerp Belgium; ^2^ Flow Cytometry and Sorting Core Facility (FACSUA) University of Antwerp Antwerp Belgium

**Keywords:** brain‐derived neurotrophic factor, mRNA electroporation, regulatory T cells, T‐cell engineering

## Abstract

Genetic engineering of regulatory T cells (Tregs) presents a promising avenue for advancing immunotherapeutic strategies, particularly in autoimmune diseases and transplantation. This study explores the modification of Tregs via mRNA electroporation, investigating the influence of T‐cell activation status on transfection efficiency, phenotype, and functionality. For this CD45RA^+^ Tregs were isolated, expanded, and modified to overexpress brain‐derived neurotrophic factor (BDNF). Kinetics of BDNF expression and secretion were explored. Treg activation state was assessed by checking the expression of activation markers CD69, CD71, and CD137. Our findings show that only activated Tregs secrete BDNF post‐genetic engineering, even though both activated and resting Tregs express BDNF intracellularly. Notably, the mTOR pathway and CD137 are implicated in the regulation of protein secretion in activated Tregs, indicating a complex interplay of signalling pathways. This study contributes to understanding the mechanisms governing protein expression and secretion in engineered Tregs, offering insights for optimizing cell‐based therapies and advancing immune regulation strategies.

## Graphical abstract

Following isolation and expansion, the function of Tregs can be enhanced through mRNA electroporation, resulting in protein overexpression. Our findings reveal that protein secretion post‐electroporation is dependent on the activation state of the Tregs. Specifically, only activated Tregs were capable of successfully secreting BDNF after BDNF‐mRNA electroporation.

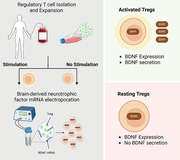

## 1 Introduction

In recent years, the advent of genetic engineering techniques has revolutionised the field of immunotherapy, offering promising avenues for the development of novel treatment modalities for various diseases, including autoimmune disorders and cancer. Among the various cell types targeted for genetic modification, regulatory T cells (Tregs) have emerged as particularly attractive candidates for genetic modification, especially for autoimmune diseases and the prevention of transplant rejection. They play a key role in maintaining immune homeostasis and tolerance, preventing autoimmunity. [1, 2]. Presently, the administration of both autologous and allogeneic Tregs is undergoing extensive testing in phase 1 and 2 clinical trials for autoimmune‐related disorders such as type 1 diabetes [3, 4], systemic lupus erythematosus [5], Crohn's disease [6], graft‐versus‐host disease [7, 8, 9], kidney and liver transplant recipients [10, 11], and multiple sclerosis (MS) [12]. Encouragingly, the reported studies have not documented serious adverse events associated with Treg administration, underscoring the safety and feasibility of this therapeutic approach [13]. Moreover, several phase 1/2 studies have included exploratory outcomes in their analyses, providing proof‐of‐concept of the use of Tregs as a therapeutic approach. The administration of Tregs resulted in modest improvements in clinical outcomes and favourable immunological effects, such as a reduction of proinflammatory cytokine production and a decrease in the number of cytotoxic CD8^+^ cells. [5, 6, 7, 14, 15, 16]. To enhance the clinical impact of polyclonal Tregs, a proposed strategy involves engineering them to execute their activity more specifically. This may entail incorporating antigen‐specific T cell receptors (TCRs) or chimeric antigen receptors (CARs) [17], but the efficacy of Tregs can also be enhanced by upregulating or introducing the expression of anti‐inflammatory cytokines or tissue‐protective and reparative factors. This targeted enhancement of Treg functionality holds promise for ameliorating a spectrum of conditions, encompassing autoimmune disorders as well as neurodegenerative diseases.

Various methods are employed for T‐cell engineering, encompassing techniques such as viral transduction, including retroviruses and lentiviruses, and nonviral approaches including mRNA electroporation. Notably, the latter presents itself as a safer alternative for the introduction of protein expression, as indicated by the absence of insertional mutagenesis risk and a diminished potential for eliciting an immune response [18]. Furthermore, mRNA electroporation offers the advantage of achieving an enhanced, albeit transient, transfection efficiency compared with alternative methods [19, 20]. In addition, mRNA electroporation has already been proven to be safe and efficacious in both clinical and preclinical studies [21].

One critical consideration in T cell genetic engineering is the choice between resting and activated T cells as the starting population for modification. Resting T cells, characterised by a quiescent state, exhibit reduced susceptibility to activation‐induced cell death and are amenable to ex vivo expansion and manipulation [22, 23]. Previous studies employing mRNA electroporation on resting lymphocytes yielded successful outcomes [24, 25]. However, the genetic modification efficiency of resting T cells via mRNA electroporation may be limited due to their low metabolic activity and reduced protein synthesis machinery [26]. Indeed, activated T cells, stimulated through T cell receptor engagement or cytokine signalling, display heightened metabolic and biosynthetic activity, making them more permissive to genetic modification. Additionally, activated T cells exhibit enhanced proliferative capacity, facilitating the expansion of engineered T cell populations for therapeutic applications [27]. However, the activation process itself may induce phenotypic and functional changes in T cells, potentially altering their regulatory properties and therapeutic efficacy [28]. Moreover, the secretion of therapeutic proteins, such as cytokines or immune checkpoint inhibitors, by genetically engineered T cells poses additional challenges. Resting T cells typically exhibit limited secretory capacity, with certain secretory pathways being less active compared with activated counterparts [26, 29]. Thus, the choice of T cell activation state may impact the secretion kinetics and efficiency of therapeutic protein production, influencing the overall therapeutic outcome.

In this study, we investigate the genetic engineering of Tregs using mRNA electroporation. Tregs will be engineered with the protein Brain‐derived neurotrophic factor (BDNF) which is a member of the neurotrophin family that plays a multifaced role encompassing synaptic modulation, axonal plasticity, neuronal survival, and facilitation of oligodendrocyte proliferation and myelination processes [30, 31, 32, 33]. Emerging evidence suggests that BDNF exhibits a neurorestorative capacity subsequent to demyelinating events [34, 35, 36, 37]. Furthermore, BDNF has notable anti‐inflammatory properties. Recent evidence suggests that BDNF regulates inflammatory homeostasis, reducing inflammatory activity through the hedgehog and erythropoietin signalling pathways [38]. By binding to microglial cells through the TrkB receptor, BDNF significantly reduces the production of proinflammatory cytokines, such as interleukin‐6 (IL‐6) and tumour necrosis factor‐alpha (TNF‐α), via multiple signalling pathways [38, 39, 40, 41]. This action is crucial for regulating immune homeostasis and controlling neuroinflammation [42]. BDNF signalling can modulate key pro‐inflammatory transcription factors, such as NF‐kB and AP‐1, and limit the inflammatory response, but the exact regulatory mechanisms of this interaction are not understood yet [40, 42, 43]. Interestingly, pro‐inflammatory cytokine stimulation downregulates BDNF expression in the hippocampus and cerebral cortex of mice [44, 45], the overexpression of BDNF in the hippocampus can mitigate synaptic dysfunction and improve neuroinflammation [41]. This demonstrates bi‐directional communication between BDNF and immune signalling and highlights the impact of inflammatory pathogenesis on neuronal homeostasis. Consequently, BDNF has been proposed as a therapeutic option for neurodegenerative and inflammatory disorders. Moreover, we explore the implications of T‐cell activation status on transfection efficiency and phenotype, aiming to optimise the design and implementation of Treg‐based immunotherapies for clinical translation.

## 2 Materials and Methods

### 2.1 Human Blood Samples and Ethical Statement

Blood samples were obtained from anonymous healthy donors and provided by the Blood Service of the Flemish Red Cross (Mechelen, Belgium). The study was approved by the Ethics Committee of the Antwerp University Hospital and Antwerp University (Belgium) under reference number 5513.

### 2.2 Isolation of CD45RA^+^ Tregs

Peripheral blood mononuclear cells (PBMCs) were separated from whole blood using density gradient centrifugation (Ficoll‐Paque PLUS, GE Healthcare, Diegem, Belgium). Following isolation, CD4^+^ cells were positively selected from 500 × 10^6^ PBMCs utilizing human CD4 MicroBeads for magnetic‐activated cell sorting (MACS, Miltenyi Biotec, Leiden, the Netherlands). Subsequently, the isolated CD4^+^ underwent staining for flow cytometric sorting, as previously described [46]. In brief, CD4^+^ cells were stained with fluorochrome‐conjugated monoclonal antibodies (mAbs) (Table S1). The CD45RA^+^ Treg population was sorted as CD3^+^, CD4^+^, CD127, CD25^+^, and CD45RA^+^. The remaining CD8^+^ T cells, monocytes, natural killer (NK) cells, and B cells were excluded from the sorting gate using a dump channel consisting of anti‐CD8, anti‐CD14, anti‐CD16, and anti‐CD19 (Figure 2A). Flow‐cytometric associated cell sorting (FACS) was performed using the FACSAria II device (BD Biosciences, Erembodegem, Belgium). Finally, an aliquot of the sorted cells was used to confirm purity using the same gating strategy.

### 2.3 Ex Vivo Expansion of Tregs

Tregs were expanded ex vivo for 14 days, as previously described [46]. For this, Tegs were suspended at a concentration of 1.5 × 10^6^ cells/mL in Iscove's modified Dulbecco's medium (IMDM; Life Technologies) supplemented with 5% human AB serum (Life Technologies) and 500 IU/mL interleukin‐2 (IL‐2; ImmunoTools). Activation of Tregs was induced by the addition of T cell TransAct (Miltenyi Biotec) which is a colloidal polymer containing anti‐CD3 and anti‐CD28 to mimic immune activation of T cells enhancing their proliferation and thus the ex vivo expansion. T cell TransAct was added at a 1:100 dilution on day 1, with subsequent reactivation on day 7. For the conditions including activated Tregs, a final restimulation was performed on day 14. The complete medium was replenished every two or three days, and cell counts were monitored using an automated hemocytometer (ABX Micros 60; Horiba, Lier, Belgium). Purity assessments on days 7 and 14 were conducted by measuring an aliquot of 100.000 events. FOXP3^+^, CD3^+^, CD4^+^, CD127^−^, and CD25^+^ cells were considered Tregs. The following fluorochrome‐conjugated mAbs were used: CD3, CD4, CD25, CD127, and FOXP3 (Table 1). Viability was determined utilizing the LIVE/DEAD Fixable Aqua Dead Cell Stain Kit (ThermoFisher).

### 2.4 Vector Construction and In Vitro mRNA Transcription

The 741 base pair sequence of BDNF was cloned into the SpeI‐XhoI site of the pST1 DNA plasmid. This cloning was executed using a T7 promoter, featuring the addition of a poly(A) tail, and subsequently underwent codon optimization (Geneart, Thermo Fisher Scientific, Regensburg, Germany; Figure 3). SoloPack Golden supercompetent *E. coli* cells were transformed with the pST1 DNA plasmids according to the manufacturer's instructions. After transformation, plasmid DNA isolation and purification were accomplished using a NucleoBond Xtra Midi EF kit (Machery‐Nagel). Following purification, plasmid DNA was linearised utilizing the SapI restriction enzyme (Thermo Fisher Scientific). Subsequently, the linearised DNA served as a template for in vitro transcription by means of a mMessage mMachine T7 in vitro transcription kit (Ambion, Life Technologies), following the manufacturer's instructions. mRNA was purified and precipitated using lithium chloride. The quality of the transcribed mRNA was assessed through agarose gel electrophoresis and Nanodrop analysis (Thermo Fischer Scientific). The ratio of absorbance at 260 and 280 nm had to be higher than 1.9 before the mRNA was considered pure. DNA plasmids (0.5 µg/µL), linearised DNA (0.5 µg/µL), and mRNA constructs (1 µg/µL) were stored at −20°C for further use.

### 2.5 mRNA Electroporation

**FIGURE 1:**
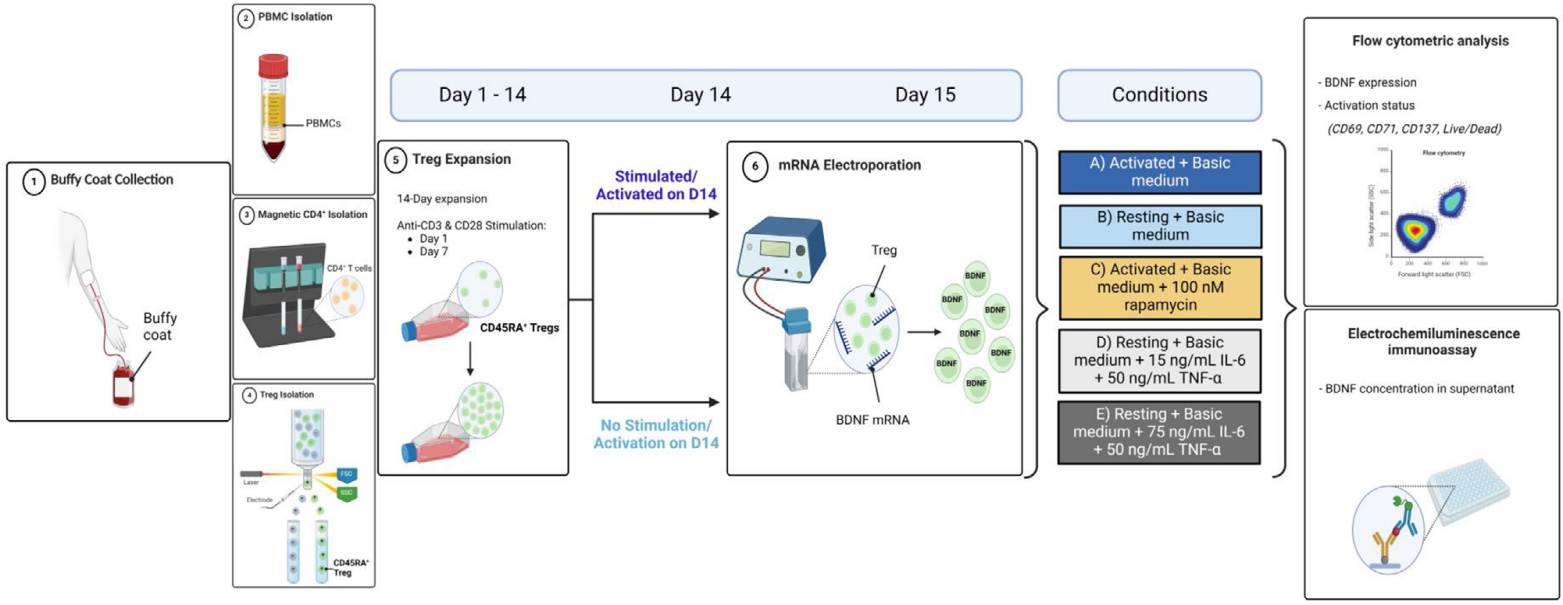
Overview of the experimental set‐up: (1) first, PBMCs were isolated from buffy coats by means of a (2) density gradient centrifugation followed by (3) positive CD4^+^ selection and (4) FACS sorting to obtain the CD45RA^+^ Treg subpopulation. (5) Next, Tregs were expanded during a 14‐day period. Tregs were stimulated by means of anti‐CD3 and anti‐CD28 activation on day 1. Restimulation was performed on day 7 for all conditions and on day 14 for the activated cells. On day 15 of the expansion, Tregs were engineered to overexpress BDNF using mRNA electroporation (6). After electroporation activated (A) and resting (B) 200,000 Tregs were cultured in 200 µL basic medium (IMDM + 500 IU/mL IL‐2) supplemented, depending on the condition, with rapamycin (C) or IL‐6 and TNF‐α (D, E). Finally, the expression pattern of BDNF and activation markers CD69, CD71, and CD137 were determined using flow cytometric analysis. The BDNF‐secretion pattern was analysed utilizing an electrochemiluminescence assay (created with Biorender).

At day 15 of cell expansion, Tregs were harvested and electroporated with mRNA encoding BDNF. For this, 5–20 × 10^6^ Tregs were harvested, washed twice, and resuspended in 200 µL of cold Opti‐MEM I medium (Gibco Invitrogen). Next, 1 µg of mRNA per 1 × 10^6^ cells were added and thoroughly mixed before being transferred to a 4.0 mm electroporation cuvette (Cell Projects). Electroporations were performed using a Gene Pulser Xcell device (Bio‐Rad) employing a square wave pulse of 500 V for 5 ms [24, 25]. As a negative control, cells underwent electroporation under identical conditions without the addition of mRNA, that is, mock electroporation. Immediately post‐electroporation, cells were resuspended in prewarmed IMDM supplemented with 10% human AB serum. After a resting period of 30 min at room temperature, cells were washed twice in phosphate‐buffered saline (PBS, Gibco Invitrogen). Subsequently, an aliquot from each experimental condition was obtained to assess cell number using an automated hemocytometer (ABX Micros 60; Horiba). Next, phenotype was assessed using CD3, CD4, CD25, and CD127 mAbs and viability was assessed utilizing the LIVE/DEAD Fixable Aqua Dead Cell Stain Kit. For this 10,000 events were measured for flow cytometric analysis (Novocyte Quanteon; Agilent, Santa Clara, USA). After electroporation, Tregs were resuspended in IMDM supplemented with 500 IU/mL IL‐2 to achieve a precise concentration of 1 × 10^6^ cells/mL. For the experiments determining the effect of the mTOR inhibitor on the BDNF secretion and expression of activated Tregs, 100 mM of rapamycin was added (Figure 1). To determine the effect of mTOR activators IL‐6 and TNF‐α on the BDNF expression and secretion on resting Tregs, 15 or 75 ng/mL IL‐6 and 50 ng/mL TNF‐α was added to the medium (Figure 1).

### 2.6 Detection of Treg Activation Markers

Tregs were harvested at different timepoints ranging from 6 to 120 h and subsequently washed twice in PBS. Next Tregs were stained with mABs against CD69, CD71 and CD137 (Table S1) and the LIVE/DEAD Fixable Aqua Dead Cell Stain Kit. Next, cells were washed twice in a staining buffer and subsequently analysed using flow cytometric analysis (Novocyte Quanteon; Agilent).

### 2.7 Intracellular BDNF Detection

**FIGURE 2:**
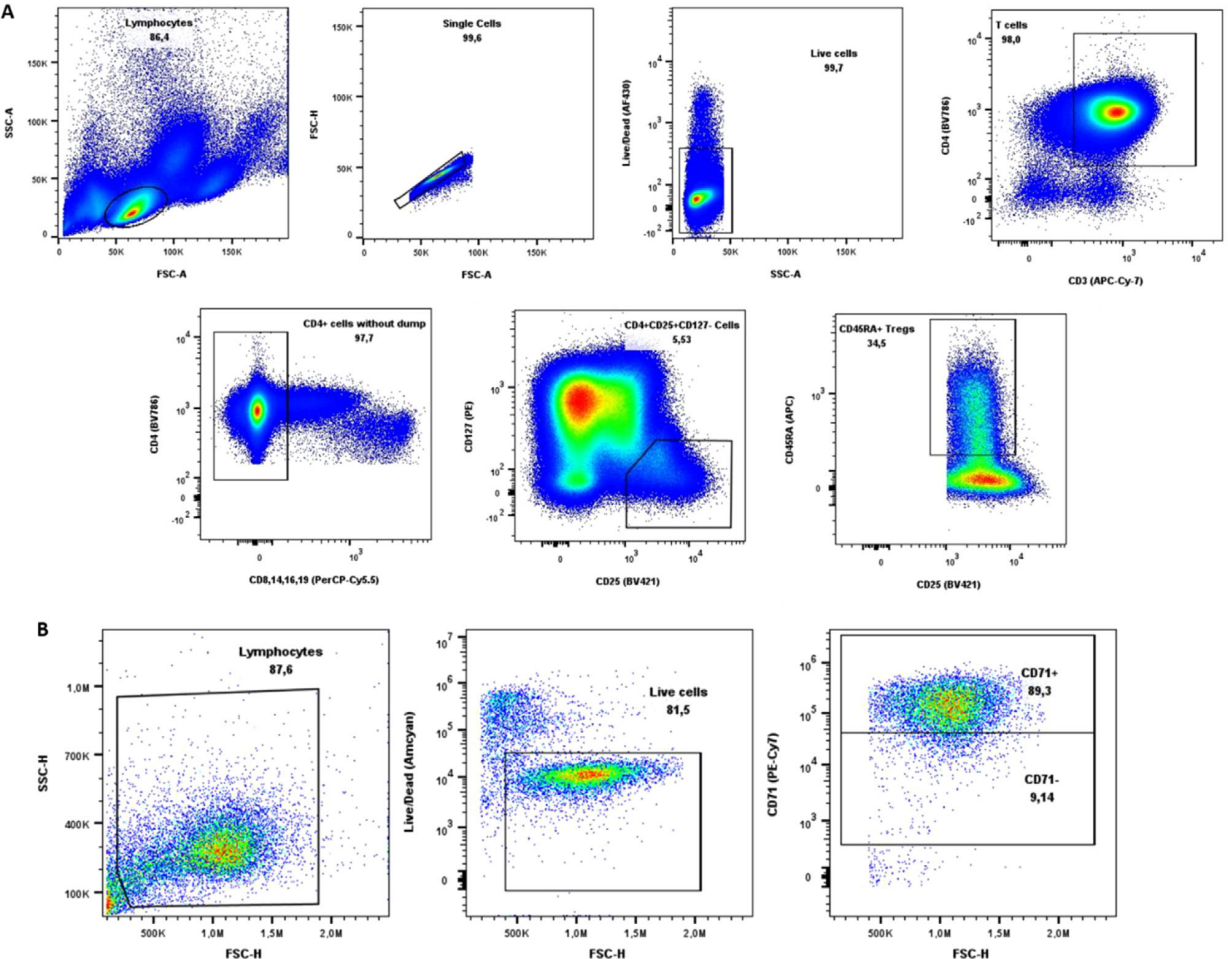
Gating strategies. (A) Gating strategy used for flow cytometric cell sorting and phenotyping of CD45RA+ Tregs after CD4^+^ magnetic bead enrichment from PBMCs. First, lymphocytes (SSC‐A/FSC‐A), single cells (FSH‐H/FSC‐A), and living cells (LIVE/DEAD fixable aqua dead cell stain negative population) were selected. Subsequently, T cells (CD3^+^ CD4^+^) were selected followed by subsequent elimination of irrelevant Tregs based on a dump channel (CD8^−^ CD14^−^ CD16^−^ CD19^−^). Treg selection was done based on CD25^+^ and CD127^−^ selection. Finally, the Treg subpopulation of interest was obtained through the selection of CD45RA^+^ cells. (B) Gating strategy to determine quantitatively the amount of translated BDNF. Cells were selected, from left to right, as lymphocytes (FSC‐H/SSC‐H), living cells (LIVE/DEAD Fixable Aqua Dead Cell Stain negative population), and as CD71^+^ or CD71^−^. From these populations, the MFI value for BDNF was determined.

Tregs were harvested at different timepoints ranging from 6 to 120 h and subsequently washed twice in PBS. Next, Tregs were fixed according to the manufacturer's protocol using eBioscience IC Fixation Buffer (Thermo Fisher Scientific) and washed twice in eBioscience Permeabilization Buffer (Thermo Fisher Scientific). After this, an anti‐BDNF mAB was added and cells were washed twice in permeabilisation buffer, and the LIVE/DEAD Fixable Aqua Dead Cell Stain Kit was added (Table S1). Finally, cells were washed twice in a staining buffer containing sheath, 1% bovine albumin serum, and 0.5% NaN_3_ and subsequently analysed using flow cytometric analysis (Novocyte Quanteon; Agilent). Transfection efficiency is quantified by determining the percentage of cells expressing BDNF, as determined within the live cell population. The quantitative assessment of translated BDNF relies on the mean fluorescence intensity (MFI) value, specifically confined to subpopulations characterised by the presence or absence of the activation markers CD71 and CD137, as illustrated in the gating strategy depicted in Figure 2B.

**FIGURE 3:**
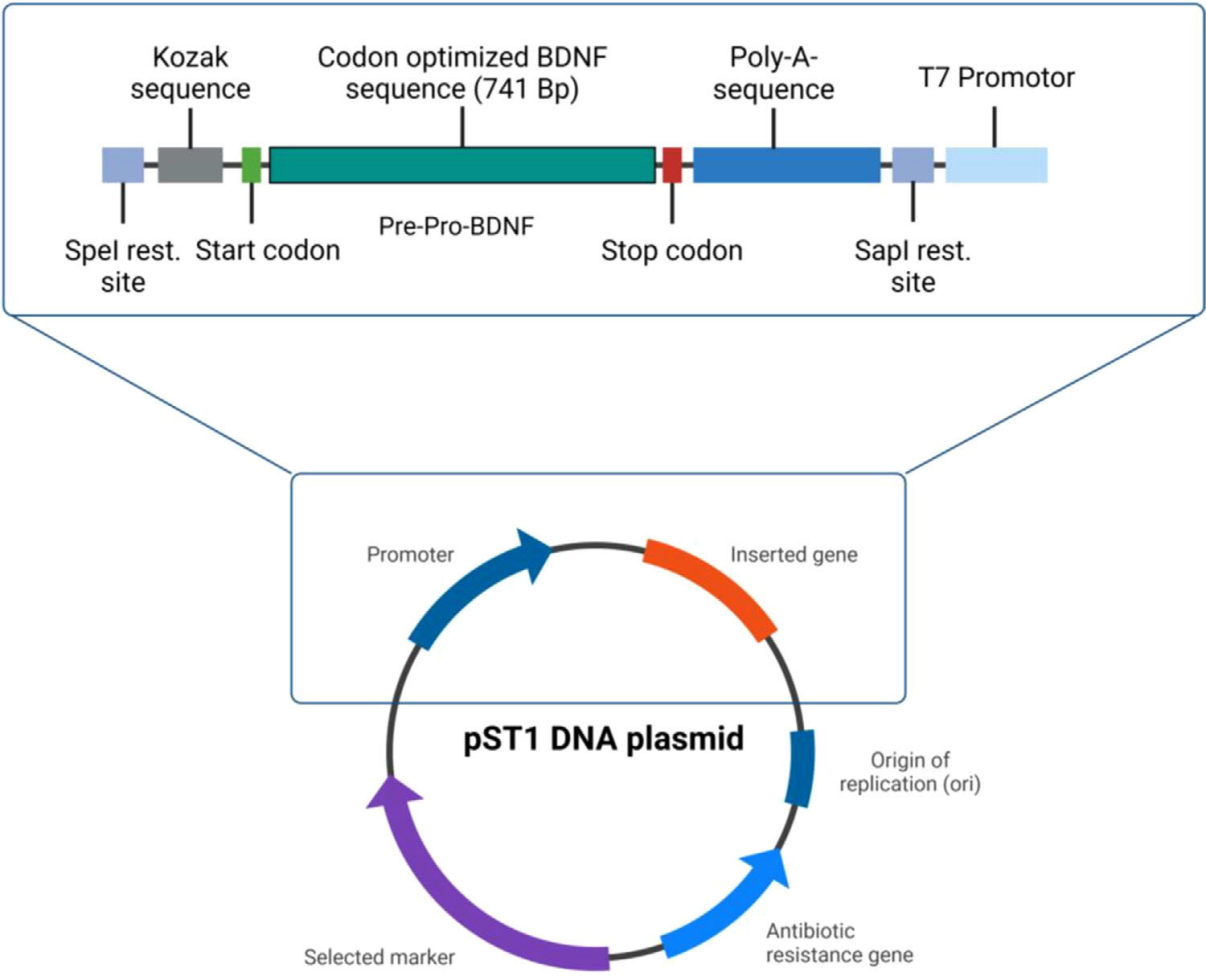
pST1 DNA plasmid containing the human and codon‐optimised BDNF sequence. The 741 base pair sequence of BDNF was cloned into the SpeI‐XhoI site of the pST1 DNA plasmid. This cloning was executed using a T7 promoter, featuring the addition of a poly(A) tail, and subsequently underwent codon optimisation. The plasmid was amplified in *E. coli* cells after transformation with the pST1 DNA plasmids. After transformation, plasmid DNA isolation and purification were performed. Following purification, plasmid DNA was linearised utilising the SapI restriction enzyme. Linearised DNA served as a template for in vitro transcription to mRNA. Finally, mRNA was purified, precipitated, and cryopreserved awaiting its use for electroporation. After electroporation, the mRNA was translated as pre‐pro‐BDNF. Subsequently, pre‐pro‐BDNF was cleaved in the Golgi‐apparatus into pro‐BDNF and mature BDNF and finally secreted into the supernatant.

### 2.8 Detection of Secreted BDNF in the Supernatant

To determine the concentration of secreted BDNF, supernatant was collected at 24 h intervals during a period of 7 days. For this, the 96‐well plate in which every well contains 200,000 cells in 200 µL was centrifuged (1544 rpm, 5 min) after which the supernatant was collected and immediately cryopreserved at –20°C. The Tregs were immediately resuspended in the correct culture medium. Finally, BDNF concentration was determined by means of an electrochemiluminescence assay (U‐PLEX Human BDNF Assay, Mesoscale Discovery; Rockville, USA), according to the manufacturer's protocol.

### 2.9 Data Analysis

FACS data were analysed using FLowJo software version 10.10.0 (BD, Biosciences). Results were analysed using Graphpad Prism software version 10 (Graphpad). To determine whether parametric or nonparametric analysis should be performed, the Shapiro–Wilk normality test and Kolmogorov–Smirnov test were performed. Statistical analysis was performed using a parametric paired *t*‐test, nonparametric Wilcoxon test, nonparametric Friedman test with Dunn's multiple comparisons test, and two‐way ANOVA or mixed‐effects analysis with the Geisser–Greenhouse correction and Sidák's multiple comparison test. A *p*‐value ≤0.05 was considered statistically significant. Normally and not normally distributed data were presented as mean ± SD and as median and interquartile range, respectively.

## 3 Results

### 3.1 Expansion of FACS‐Isolated Tregs Resulted in a >75‐Fold Increase While Preserving the Treg Phenotype

**FIGURE 4:**
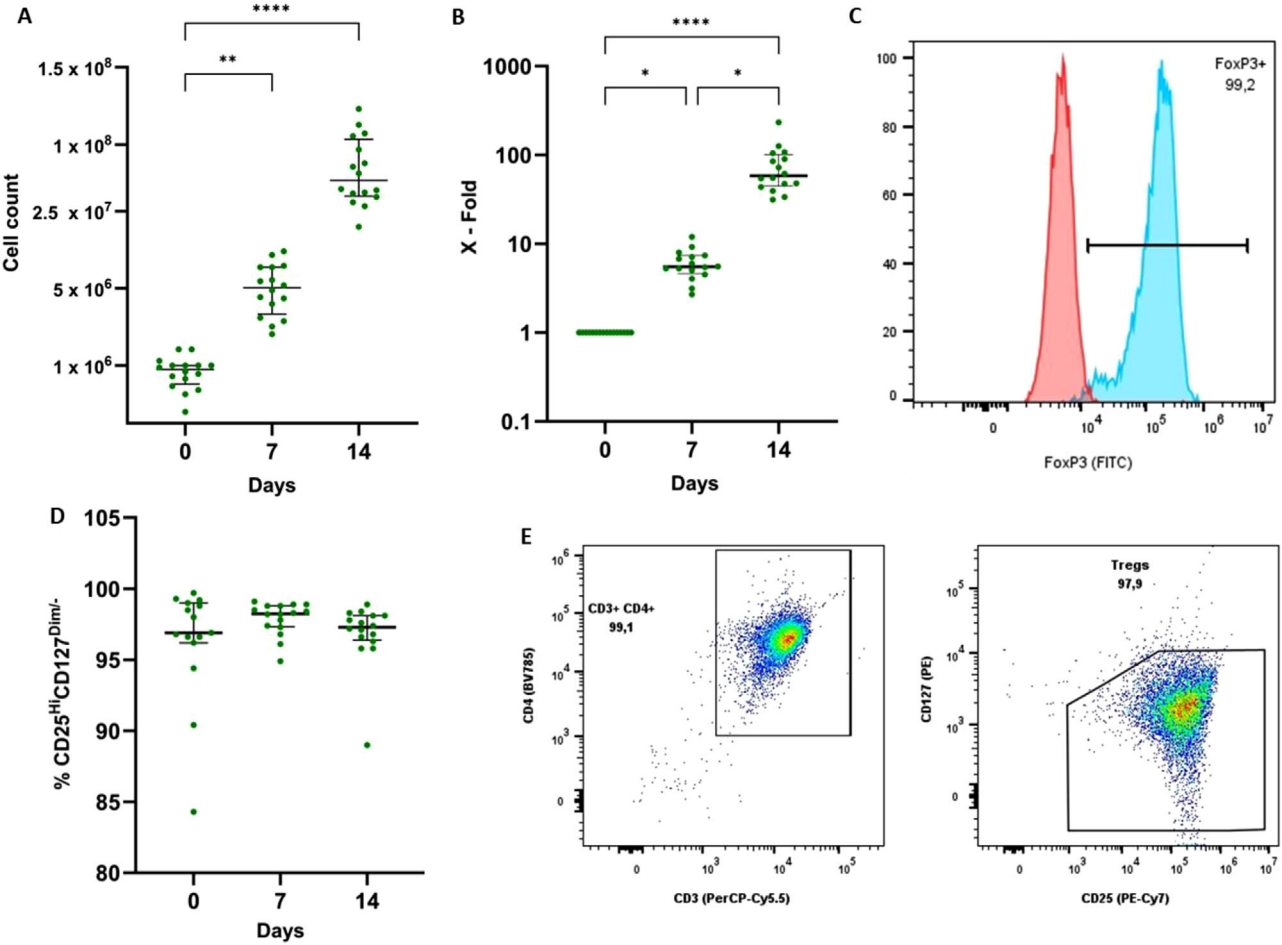
Isolation and expansion of the CD45RA^+^ Treg population. (A) Fourteen‐day expansion showed a significant increase in the starting population both after 7 (*p* = 0.0037) and 14 days (*p* < 0.0001). (B) After 7 days a significant fold increase (*p* = 0.0037) was observed. After 14 days a significant fold‐expansion was observed compared with day 0 (*p* < 0.0001) and day 7 (*p* = 0.0140). (C) Representative example of the histogram overlay plot used to determine the FOXP3 expression in expanded Tregs. PBMC samples were used as a biological negative control. (D) Treg purity was assessed using flow cytometry. CD3^+^ CD4^+^ CD127^Dim/‐^ CD25^High^ were considered as Tregs. No differences in Treg characterising markers were observed during expansion. (E) Representative dot plots were used to determine Treg purity. Results are shown as median ± interquartile range for 16 independent donors. Statistical analysis was performed using the nonparametric Friedman test with Dunn's multiple comparisons test: *p < 0.05, **p < 0.01, ****p < 0.0001.

Initially, the CD4^+^CD127^−^CD25^+^CD45RA^+^ Treg subpopulation was isolated using FACS. On average, a Treg purity of 97.30% [96.38–98.10%] (*n* = 16) was obtained following flow cytometric cell sorting of Tregs. Given the frequency of the Treg subpopulation of interest (2.69 [1.57–4.5%] of the CD4^+^ cells) and the number of cells post‐isolation (0.92 [0.68–1.00) × 10^6^ cells), an expansion was imperative before these cells could be used for further experiments. Therefore, an expansion protocol was used to significantly increase the number of Tregs. We observed a significant increase in Tregs, as compared with day 0, after 7 days of expansion (5.51 [4.63–7.34] × 10^6^ cells, *p* = 0.0037) and after 14 days of expansion (58.44 [44.69–111.80] × 10^6^ cells, *p* < 0.0001) (Figure 4A). A 5.05 [2.93–7.77]‐fold was observed on day 7, increasing to a 47.30 [34.20–111.8]‐fold expansion by day 14 (Figure 4B). Assessment of the Treg surface markers CD3, CD4, CD25, and CD127 showed that a pure population of Tregs (98.80% [97.33–98.80%] at day 7 and 97.30% [96.38–98.10%] at day 14) was expanded without contamination of CD127^+^ effector T cells (Figure 4D). Next, an assessment of the FOXP3 expression throughout the expansion process was conducted. Here, we observed an expression of 94.55% [91.05–97.50%] and 91.10% [88.75–94.90] on day 7 and 14, respectively (Figure 4C). Viability assessment reported a viability of 93.30% [88.65–95.35%] on day 7, and of 93.35% [91.20–95.98%] on day 14.

### 3.2 Electroporation of Expanded Tregs With BDNF‐Encoding mRNA Results in a High Expression and Secretion of BDNF Protein

**FIGURE 5:**
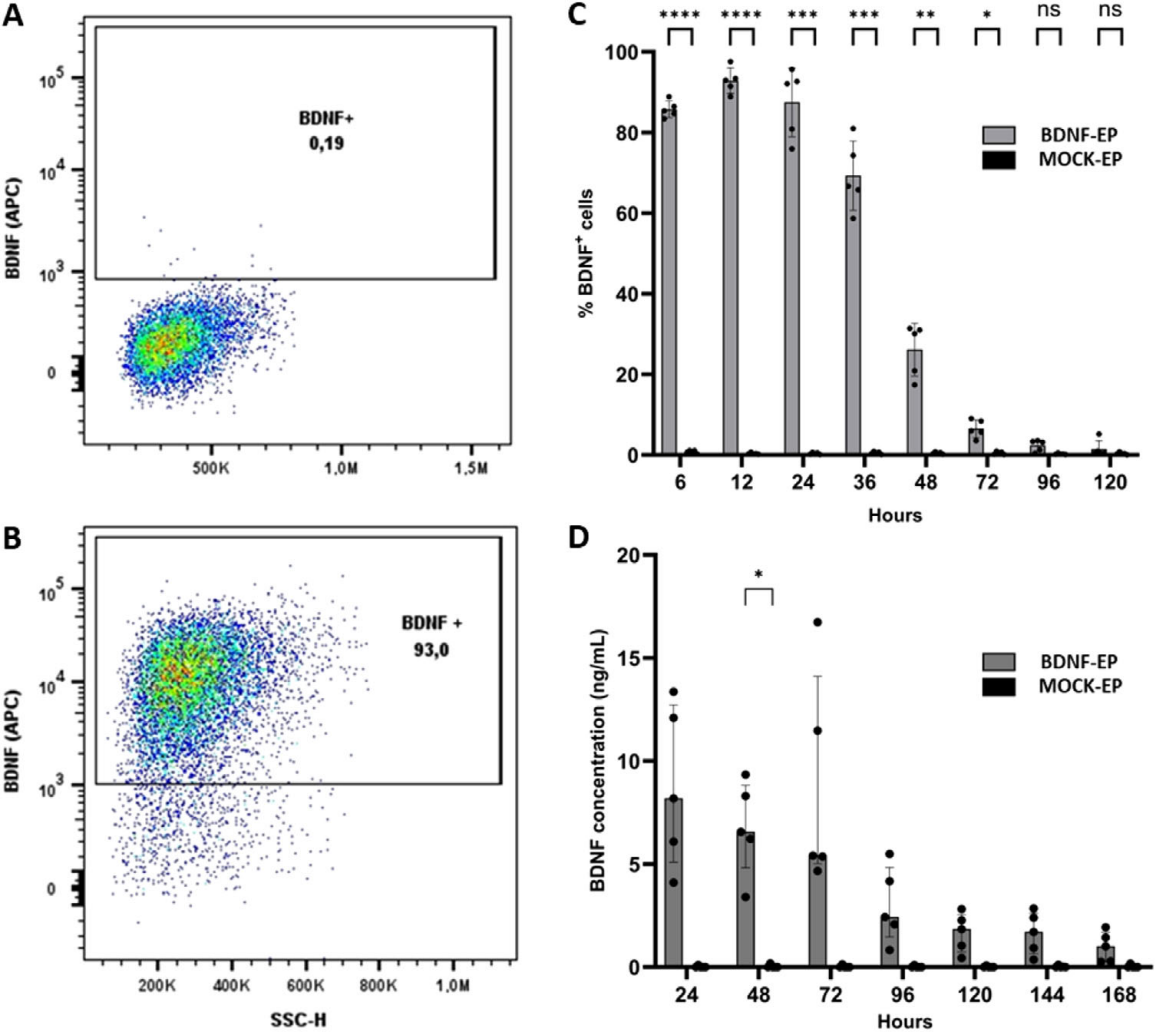
Expanded Tregs were able to successfully express and secrete BDNF after mRNA electroporation. (A) Representative dot plot showing BDNF^+^ Tregs 12 h after mock electroporation, which was used to determine the gating strategy. (B) 12 h after BDNF electroporation a clear BDNF^+^ population was observed. (C) Kinetics of the BDNF expression after BDNF or mock electroporation. (D) Comparative analysis of the secretory pattern of BDNF between BDNF and mock electroporated cells. Results are shown as a median and interquartile range for five independent donors. Statistical analysis was performed using two‐way ANOVA with the Geisser–Greenhouse correction and Sidák's multiple comparison: *p < 0.05, **p < 0.01, ***p < 0.001, ****p < 0.0001.

Next, we investigated the possibility of genetically modifying the expanded Tregs to express and secrete BDNF. For this, electroporation with BDNF‐encoding mRNA of the Tregs was performed. A successful transfection and translation of the BDNF mRNA into the BDNF protein was observed, as indicated by 93.0% [90.2–95.5%) BDNF‐expressing Tregs observed 12 h after electroporation (Figure 5B). In contrast, 0.33% [0.25–0.53%] of BDNF‐expressing Tregs was found in the mock electroporated cells (Figure 5A). The highest percentage of cytoplasmic BDNF expression was observed 12 h post‐electroporation and exhibited a gradual decline until convergence was observed between the BDNF and mock electroporated cells, 96 h post‐electroporation (Figure 5C). Interestingly, secretion of the BDNF protein persisted up to 7 days post‐electroporation compared with mock electroporated cells. Peak BDNF secretion occurred during the initial 3 days, with a maximal concentration of 8.19 [5.10–12.74] ng/mL after 24 h. Subsequently, the secreted BDNF gradually decreased over time (Figure 5D). In contrast, very low concentrations (0.00 [0.00–0.05] ng/mL) of secreted BDNF were detected in the negative control, that is, mock‐electroporated cells. Viability was assessed 24 h after mRNA electroporation for all 16 donors and these results showed a decreased viability (79.69 ± 11.25%) when compared with expanded Tregs prior to electroporation (96.36 ± 2.09; Figure S2).

### 3.3 Activated Tregs Showed a Significantly Higher BDNF Secretion Compared With Resting Tregs

**FIGURE 6:**
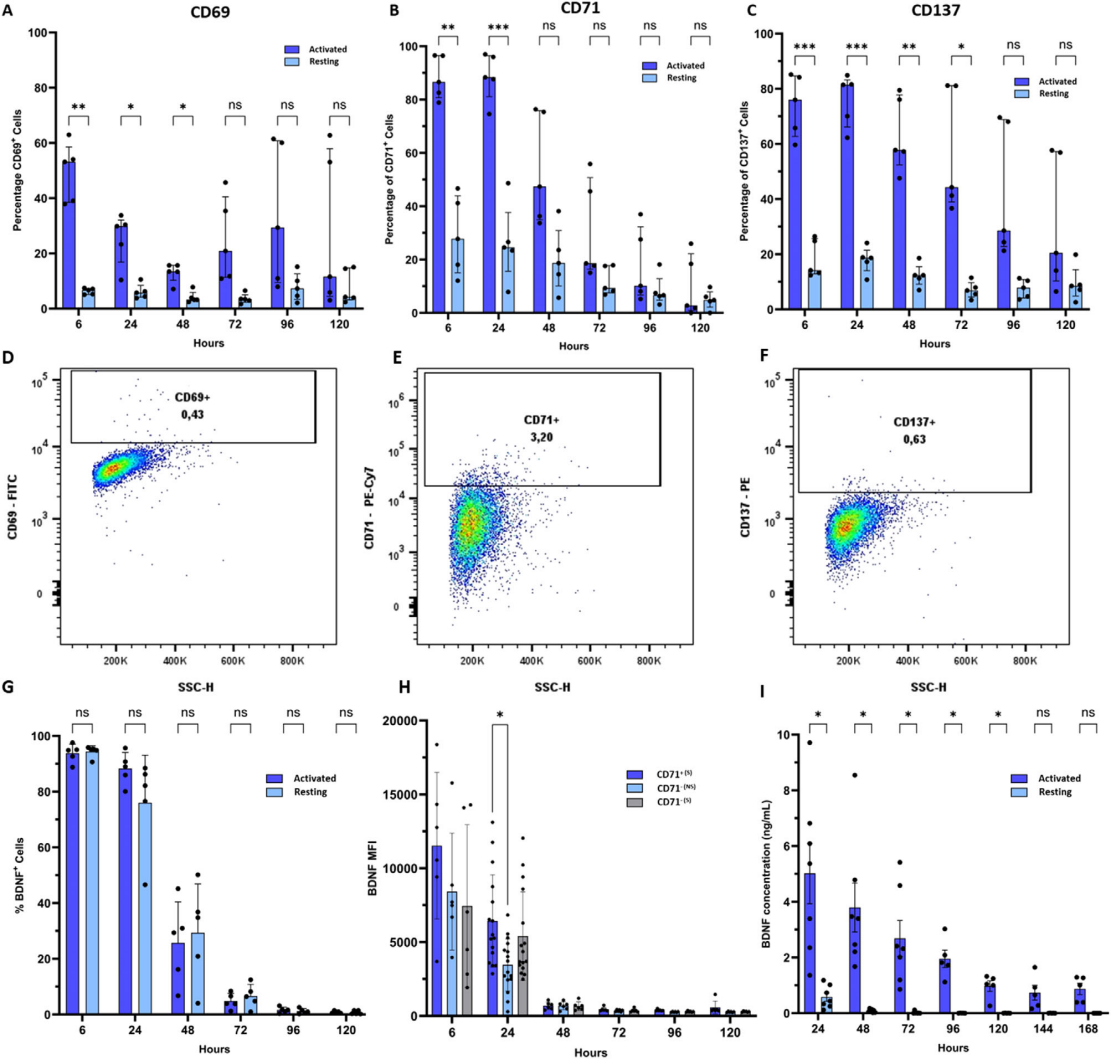
Activated Tregs secrete significantly higher concentrations of BDNF as compared with resting Tregs following mRNA electroporation. (A–C) Percentage of the cells with positive expression for activation markers CD69, CD71 and CD137 over time, for both activated (dark blue) and resting (light blue) Tregs after mRNA electroporation. (D–F) These dot plots show the biological negative control that was used to determine the gating strategy. (G) Percentage of BDNF^+^ cells after mRNA electroporation in activated and resting Tregs. (H) Histogram plot comparing the MFI value for BDNF between activated CD71^+^ cells (dark blue), activated CD71^−^ (grey), and resting CD71^−^ cells (light blue) after electroporation. (I) BDNF secretion over time between activated and resting Tregs. Results are shown as mean ± SD for seven independent donors. Statistical analysis was performed using a two‐way ANOVA or Mixed‐effects analysis with the Geisser‐Greenhouse correction and Sidák's multiple comparison test: *p < 0.05.

Activated Tregs exhibit a significantly higher expression of the early activation marker CD69 during the first three timepoints, mid‐early activation marker CD71 for the first two timepoints, and late activation marker CD137 during the first four timepoints, 6‐, 24‐, 48‐ and 72 h post‐electroporation. Peak expressions were observed 6 h after electroporation for CD69 and 24 h after electroporation for CD71 and CD137, gradually diminishing thereafter. Conversely, resting Tregs demonstrated low expression of activation markers, which remained relatively stable over time (Figure 6A–C). Expanded Tregs which were not stimulated 10 days prior to the analysis were used as biological negative controls to determine the gating strategy (Figure 6D–F). Subsequently, a comparative evaluation of the cytoplasmic expression of BDNF over a 5‐day time course between resting and activated cells, restricted to viable cells, was performed. Viability assessment at 24 h post‐electroporation indicated notably higher viability for activated Tregs compared with resting Tregs (80.6 ± 5.9% vs. 61.0 ± 17.5%, *p* = 0.0131). Maximal BDNF expression occurred at 6 h post‐electroporation, reaching 94.9% [90.7–96.4%] for activated Tregs and 95.2% [92.8–95.6%] for resting Tregs. This expression declined progressively over time, reaching a minimal percentage of BDNF‐positive cells at 120 h post‐electroporation (1.0% [0.8–2.5%] for activated Tregs and 0.3% [0.3–1.5%] for resting Tregs) (Figure 6G). These findings indicate a similar expression pattern for activated versus resting Tregs, albeit an increase in the amount of cytoplasmatic BDNF in the CD71^+^ activated Tregs was observed as compared with CD71^−^ resting Tregs. This significant difference was only observed during the second timepoint 24 h post‐electroporation (Figure 6H). In contrast, kinetics analysis of the BDNF secretion in the supernatant of activated Tregs demonstrated a significantly higher BDNF concentration during the first 5 days post‐electroporation compared with resting Tregs. Peak secreted concentrations occurred within the initial 24 h after electroporation, reaching 5.37 [2.36–6.82] ng/mL for activated Tregs and 0.44 [0.25–1.02] ng/mL for resting Tregs. Subsequently, BDNF secretion declined, with activated Tregs exhibiting a minimal concentration on day 6 (0.28 [0.00–0.93] ng/mL and resting Tregs on day 7 (0.0 ng/mL) (Figure 6I). These findings underscore a substantial influence of the activation status on the capacity of Tregs to secrete BDNF following mRNA electroporation.

### 3.4 Protein Secretion in Tregs After Electroporation Is Partially Dependent on mTOR

**FIGURE 7:**
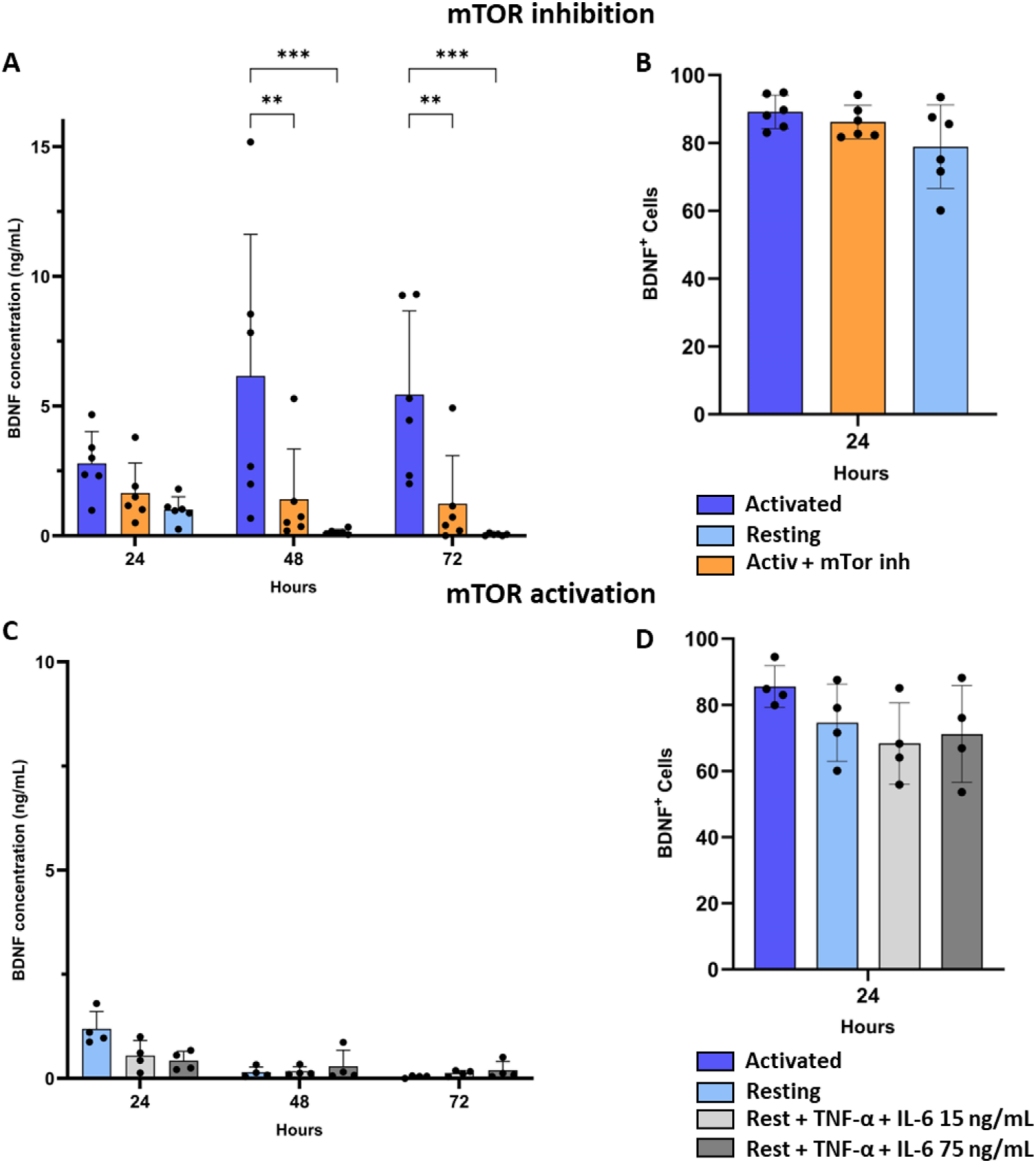
Protein secretion after mRNA electroporation is partially dependent on the mTOR pathway. (A) BDNF secretion in the different conditions, 24, 48, and 72 h post‐electroporation. A significant decrease in BDNF secretion was observed in the condition with rapamycin both for timepoints 48 and 72 (*p* = 0.0035 and *p* = 0.0107). (B) BDNF expression after mRNA electroporation for the activated cells cultured in basic medium, activated cells cultured with mTOR inhibitor rapamycin, and resting cells cultured in basic medium. (C) BDNF secretion after electroporation showed no statistical increase in secretion after the addition of mTOR activators. (D) BDNF expression after mRNA electroporation for resting and activated cells cultured in basic medium, and resting cells cultured in medium supplemented with 50 ng/mL TNF‐α and 15 ng/mL IL‐6 or 50 ng/mL TNF‐α and 75 ng/mL IL‐6. Statistical analysis was performed using the nonparametric Kruskal–Wallis test with Dunn's multiple comparisons test and a two‐way ANOVA or mixed‐effects analysis with the Geisser–Greenhouse correction and Sidák's multiple comparison tests: *p < 0.05, **p < 0.01, ***p < 0.001.

Given the difference observed in the capacity to secrete BDNF following mRNA electroporation in activated versus resting Tregs, we investigated whether this difference was dependent on the mTOR pathway. Research has already shown that mTOR plays a significant role in the (un)conventional secretion pathway of proteins [42, 43]. Therefore, we tested whether the secretion of BDNF in our engineered Tregs was mTOR dependent. For this, rapamycin was added immediately after electroporation to activate Tregs. Our data showed that BDNF secretion was significantly decreased after the addition of rapamycin. Indeed, whereas secretion remained comparable during the first day, a distinct difference was observed between the activated Tregs with and without the addition of rapamycin during the 2nd and 3rd days. After 48 h, concentration in the condition with rapamycin was reduced to 0.62 [0.31–2.32] ng/mL versus 5.26 [1.67–10.20] ng/mL in the condition without rapamycin (*p* = 0.0035). Throughout the 3rd day, concentrations of 0.56 [0.15–2.10] ng/mL and 4.88 [2.24–9.28] ng/mL were observed for the condition with and without rapamycin, respectively (*p* = 0.0107) (Figure 7A). Conversely, no differences in percentages of BDNF‐positive cells were observed in activated Tregs with and without the addition of rapamycin (Figure 7B). Next, we investigated whether we could rescue BDNF secretion in resting Tregs by activating the mTOR pathway. For this, mTOR activators TNF‐α and IL‐6 were immediately added after electroporation. We observed, however, no increase in BDNF secretion up to 3 days after electroporation when 15 ng/mL IL‐6 was added nor when 75 ng/mL IL‐6 was added (Figure 7C,D).

## 4 Discussion

The genetic engineering of Tregs holds significant promise for the development of novel immunotherapeutic strategies, particularly in the context of autoimmune diseases and transplantation [13, 17, 47]. In this study, we investigated the genetic modification of Tregs using mRNA electroporation and explored the impact of T‐cell activation status on transfection efficiency and phenotype. Our findings shed light on the complex interplay between T‐cell activation status, protein expression, and secretion, with implications for the therapeutic potential of engineered Tregs. We found that upon genetic engineering using electroporation with BDNF‐encoding mRNA in Tregs only activated Tregs are capable of secreting BDNF, albeit that both activated and resting Tregs express the BDNF protein intracellularly. This discrepancy between protein expression and protein secretion in activated versus resting Tregs seems to be partly mediated by the mTOR pathway and CD137.

Previous research has demonstrated that T‐cell activation status can profoundly influence the efficacy and functionality of genetic engineering approaches. Indeed, Smits et al. reported successful expression of the transfected reporter gene eGFP exclusively in stimulated CD4^+^ and CD8^+^ lymphocytes [48]. Also, recent observations from clinical trials indicate that stimulated T lymphocytes are essential for the successful expression of a CAR [49, 50, 51]. In contrast, Schaft et al. [24] achieved successful expression of a TCR following transfection of unstimulated CD8^+^ cells, whereas others also observed the expression and secretion of IL‐12p70 after electroporation of unstimulated PBMCs [25]. In alignment with these findings, we observed a similar transfection efficiency in activated versus resting Tregs. However, intriguingly, our results indicate that only activated Tregs are capable of secreting BDNF following genetic engineering with BDNF‐encoding mRNA. Following T‐cell activation, there is a notable upregulation of protein synthesis, peaking between 24 and 48 h post‐activation [29]. Consistently, our data exhibited a significant increase in the expression of BDNF protein in activated CD71^+^ Tregs 24 h post‐electroporation, compared with resting CD71^−^ Tregs. From these observations, we can conclude that the activation status of Tregs does not significantly impact transfection efficiency. However, it does influence the quantity of protein synthesised and subsequently secreted. Specifically, while both activated and resting Tregs exhibit comparable transfection efficiencies, activated Tregs demonstrate a higher propensity for protein synthesis and secretion compared with their resting counterparts. Although this phenomenon can be attributed to the upregulation of cellular machinery involved in mRNA translation and protein synthesis in activated T cells, our findings suggest a differential regulation of protein secretion in activated Tregs.

The discrepancy between protein expression and secretion in activated versus resting Tregs highlights the complexity of cellular signalling pathways involved in the regulation of protein trafficking and secretion. One potential mechanism underlying this phenomenon involves the mTOR pathway, a central regulator of cell growth, metabolism, and protein synthesis [52, 53]. Activation of the mTOR pathway in response to TCR signalling promotes protein translation and secretion in activated T cells, facilitating their effector functions. In contrast, resting Tregs exhibit lower mTOR activity, which may limit their capacity for protein secretion despite intracellular protein expression [54]. Notably, mTORC1 plays a pivotal role in both protein translation and secretion [55]. Indeed, a significant reduction in BDNF secretion was observed upon the addition of the mTOR inhibitor, rapamycin, to activated Tregs post‐electroporation. However, subsequent experiments introducing mTOR activators IL‐6 and TNF‐α to resting Tregs yielded contradictory findings, as there was no noticeable increase in secretion despite mTOR pathway activation. From these observations, we infer that while mTOR exerts a significant influence, it may not be the exclusive determinant governing secretion, implicating additional factors in this process. Hence, further investigations are warranted to unravel the precise mechanism underlying the expression and secretion of incorporated molecules following electroporation.

In this study, the naive CD45RA^+^ Treg subpopulation was used because of its distinct advantages, including the preservation of high immunosuppressive functionality, superior expandability compared with CD25^High^ cells, and the ability to maintain a stable Treg phenotype over the course of the expansion and electroporation [13, 46, 56]. Using our expansion protocol, we were able to initiate a substantial 47‐fold increase over a 14‐day timeframe of Tregs with the maintenance of Treg stability, phenotype, and immunosuppressive capacities [46]. The magnitude of this fold increase surpasses the requisite threshold for intrathecal administration, which typically necessitates only a few million cells [12]. By means of leukapheresis, a process enabling the isolation of up to 20 × 10^9^ PBMCs from a single donor, a substantial amount of starting material is made available [57]. Consequently, this adequate cellular reservoir furnishes sufficient material to attain a sufficiently high cell count suitable for intravenous administration which typically involves the administration of a few million cells up to 40 × 10^6^ polyclonal Tregs per kilogram [3, 12, 16, 58].

During this expansion protocol, naïve CD45RA^+^ Tregs were activated using anti‐CD3 and anti‐CD28 mimicking antigen stimulation inducing the expression of several molecules including CD69, CD71, and CD137 [59, 60, 61]. Our findings revealed a marked upregulation in the expression of the surface marker CD69, a prominent early activation marker for T cells, following stimulation. CD69 not only serves as an indicator of early T cell activation but also holds significance in the immunomodulatory function of Tregs. This underscores the imperative to induce activation in Tregs prior to their manipulation for clinical applications [62]. Furthermore, the activated state of Tregs was supported by a significant increase in the surface expression of CD71 compared with CD71 expression by their resting Treg counterparts. Additionally, our findings suggest a role for CD137, a co‐stimulatory receptor expressed on activated T cells, in modulating protein secretion in engineered Tregs (Figure S1). CD137 signalling has been implicated in the regulation of T cell activation, survival, and effector functions, and recent studies have demonstrated that inducible CD137 is a feature of killer T cells as indicated by a promoting role of CD137 for increased mitochondrial biogenesis and enhanced interferon‐gamma secretion. This potentially suggests a direct link between cytotoxicity and, hence, secretory function, and CD137, similar to CD107a and its role as a surrogate marker for degranulating T cells [63, 64, 65]. It is plausible that CD137‐mediated signalling pathways intersect with the mTOR pathway to regulate protein secretion in activated Tregs, although further mechanistic studies are warranted to elucidate the precise molecular mechanisms involved.

The implications of our findings extend beyond the field of Treg engineering and have broader implications for the design and optimization of cell‐based therapies. Understanding the factors influencing protein expression and secretion in engineered T cells is critical for enhancing therapeutic efficacy and safety. Future studies aimed at dissecting the molecular pathways governing protein secretion in different T cell subsets may uncover novel targets for therapeutic intervention and provide insights into the mechanisms of immune regulation and tolerance induction. Overall, our findings contribute to the growing body of knowledge on T cell biology and hold promise for the development of next‐generation cell‐based immunotherapies.

## Author Contributions

Conceptualization: Jasper Van den Bos, Nathalie Cools, Inez Wens, and Yousra El Ouaamari. Data collection: Jasper Van den Bos, Morgane Vermeulen, Amber Dams, and Stefanie Peeters. Experimental support: Hans De Reu and Carole Faghel. Data analysis and writing—original draft preparation: Jasper Van den Bos and Nathalie Cools. Writing—review and editing: Jasper Van den Bos, Nathalie Cools, and Inez Wens. Visualization: Jasper Van den Bos. Supervision: Nathalie Cools and Inez Wens. Funding acquisition: Jasper Van den Bos, Inez Wens, and Nathalie Cools.

## Conflicts of Interest

The authors declare no conflicts of interest.

### Peer Review

The peer review history for this article is available at https://publons.com/publon/10.1002/eji.202451005

## Supporting information



Supporting Information

Supporting Information

## Data Availability

The data that support the findings of this study are available from the corresponding author upon reasonable request.
