# Supplementary material for: The Efficiency of Brain‐Derived Neurotrophic Factor Secretion by mRNA‐Electroporated Regulatory T Cells Is Highly Impacted by Their Activation Status
Source: Eur J Immunol. 2024 Dec 19;55(2):e202451005. doi: 10.1002/eji.202451005 (PMC11830389; doi:10.1002/eji.202451005)
Supplement: Supplementary file 1 — Supporting Information [file EJI-55-e202451005-s001.docx]

| Target | Fluorochrome | Clone | Ref. Number (Company) |
| --- | --- | --- | --- |
| CD3 | APC-H7 | SK7 | 560176 (BD) |
| CD3 | PerCp-Cy5.5 | ICHT1 | 560835 (BD) |
| CD4 | BV785 | RPA-T4 | 300554 (Biolegend) |
| CD8 | PerCP-Cy5.5 | SK1 | 344710 (Biolegend) |
| CD14 | PerCP-Cy5.5 | 63D3 | 367110 (Biolegend) |
| CD16 | PerCP-Cy5.5 | 3G8 | 302028 (Biolegend) |
| CD19 | PerCP-Cy5.5 | SJ25C1 | 363016 (Biolegend) |
| CD25 | BV421 | M-A251 | 356114 (Biolegend) |
| CD25 | PE-Cy7 | M-A251 | 557741 (BD) |
| CD45RA | APC | HI100 | 304112 (Biolegend) |
| CD69 | FITC | FN50 | 555530 |
| CD71 | PE-Cy7 | CY1G4 | 334112 (Biolegend) |
| CD127 | PE | HIL-7R-M51 | 557938 (BD) |
| CD137 | PE | 4B4-1 | 555956 (BD) |
| FOXP3 | AF-488 | 259D | 320212 (Biolegend) |
| BDNF | APC | 35909 | MA5-23665 (Thermofisher) |
| LIVE/DEAD™ Fixable Aqua Dead Cell Stain Kit | 405nm excitation | / | L34957 (Thermofisher) |

**Table S1: Information of antibodies and dyes used in flow cytometry experiments conducted in this study.** For each antibody or dye used in this study, fluorochrome, clone, reference number and company name is given, when applicable.


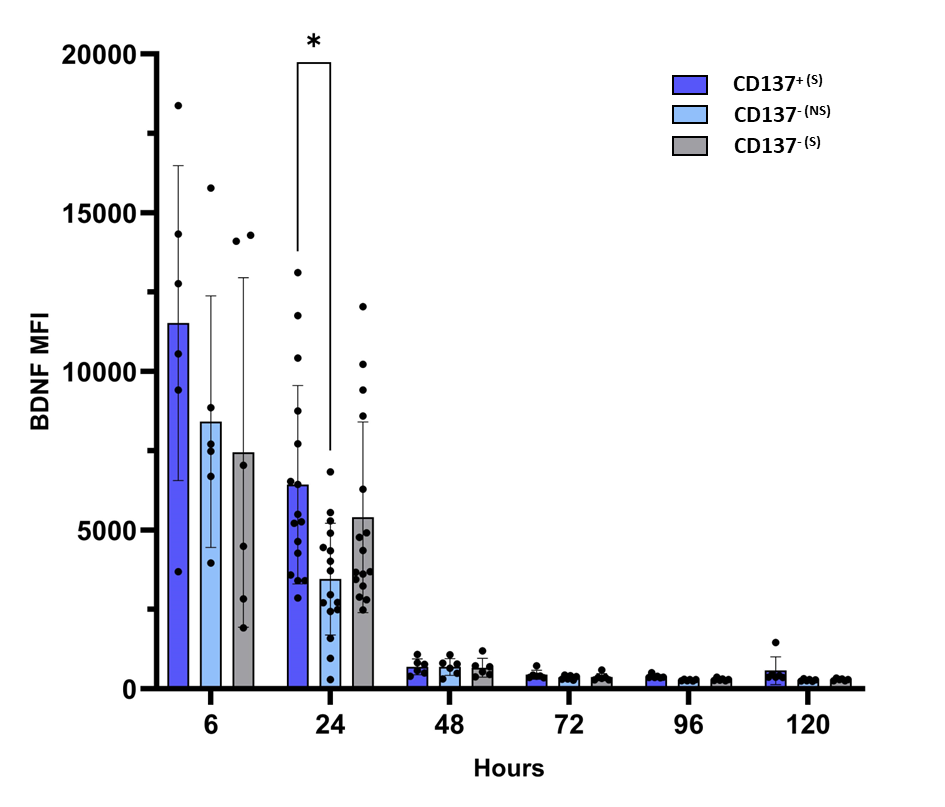


**Supplementary figure 1:** Histogram plot comparing the MFI value for BDNF between activated CD137^+^ cells (dark blue), activated CD137^-^ (grey) and resting CD137^-^ cells (light blue) after electroporation. These data suggest a role for CD137 in modulating protein secretion in engineered Tregs.
