# Supplementary material for: The Efficiency of Brain‐Derived Neurotrophic Factor Secretion by mRNA‐Electroporated Regulatory T Cells Is Highly Impacted by Their Activation Status
Source: Eur J Immunol. 2024 Dec 19;55(2):e202451005. doi: 10.1002/eji.202451005 (PMC11830389; doi:10.1002/eji.202451005)
Supplement: Supplementary file 2 — Supporting Information [file EJI-55-e202451005-s002.docx]

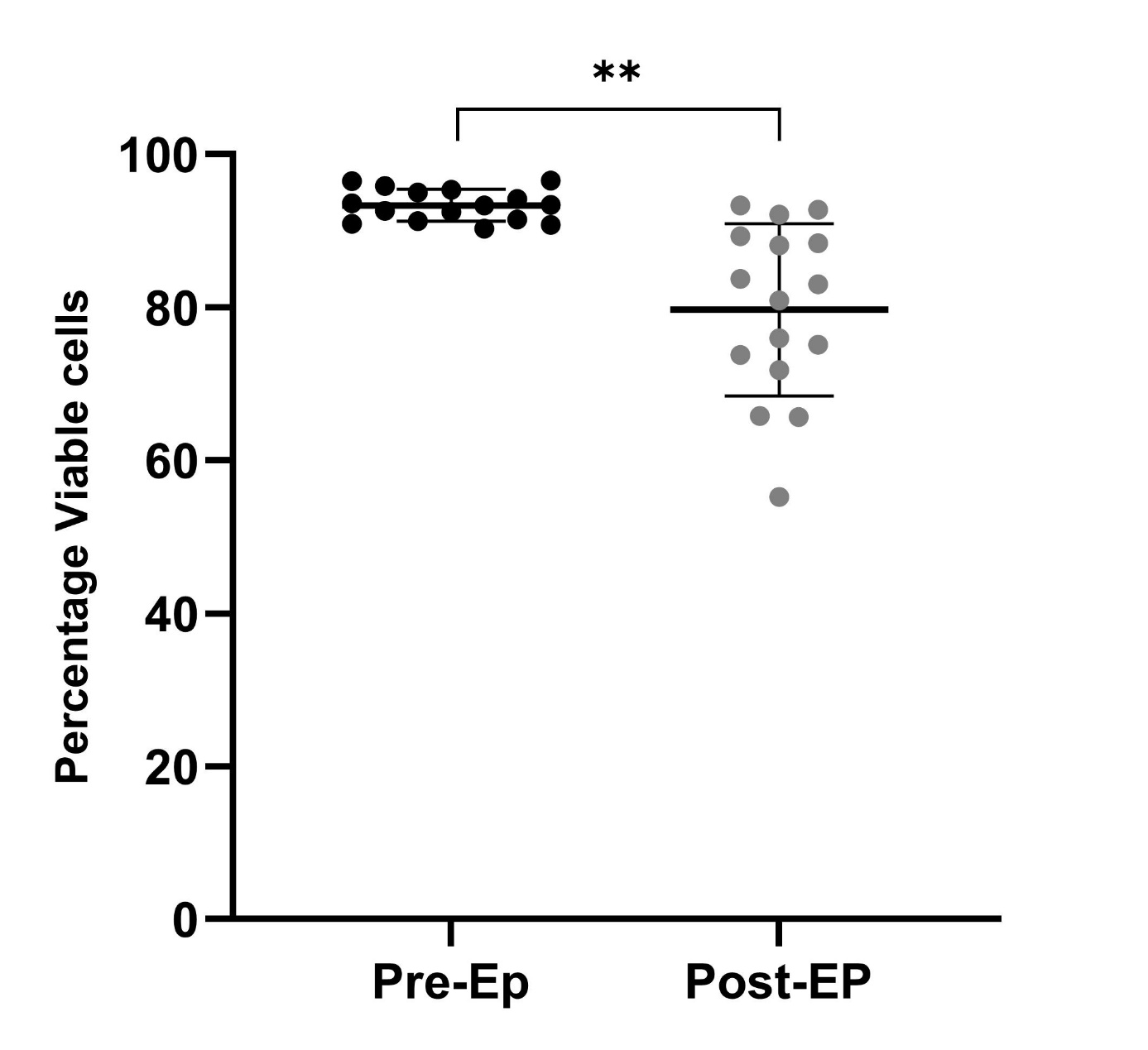


**Figure S2:** Comparison of the percentage viable cells before and 24 hours after electroporation. *Statistics were performed using paired t test: ** P < 0.01*
